# Fluid and Electric Field Simulation and Optimization of the Multi-Vane and Multi-Slit Electrospinning Nozzle

**DOI:** 10.3390/nano15060461

**Published:** 2025-03-19

**Authors:** Jian Liu, Shoujun Dong, Yongru Liu, Shanshan Pan, Zhaosong Yin

**Affiliations:** 1 School of Mechanical Engineering, Tiangong University, Tianjin 300387, China; 2231050764@tiangong.edu.cn (S.D.); 2331050787@tiangong.edu.cn (S.P.); 2331050798@tiangong.edu.cn (Z.Y.); 2 National Experimental Teaching Demonstrating Center of Engineering Training, Tiangong University, Tianjin 300387, China; 20109030@tiangong.edu.cn

**Keywords:** electrospinning, multi-vane, multi-slit, multiple jets, fluid spreading, electric field simulation

## Abstract

A multi-vane and multi-slit electrospinning nozzle for diversion was proposed to respond to the issues of easiness of clogging, existing End Effect among needles in current multi-needle electrospinning, and uncontrollable Taylor cone position in needleless electrospinning. The upper part of the novel nozzle is a cylindrical straight pipe, and the lower part is a flow channel expansion structure composed of multiple vane components that spread outward at an angle. Ansys software was used to study the effect of different opening angles of the vanes on the spreading of the electrospinning solution. In the fluid simulation, for the novel nozzle with a central slit and a support structure, when the vanes have an opening angle of 35° and a length of 11 mm, the droplet holding time is 16 s, twice as long as the nozzle without support (8 s). This result corresponds to the subsequent droplet holding experiment, showing that the support structure aids droplet holding and enhances electrospinning stability. Comsol Multiphysics software was used to investigate the effect of the vanes’ parameters on the uniformity of the electric field. The results indicate that when the vanes of the new electrospinning nozzle are set at an opening angle of 35°, with four vanes each 11 mm in length, a receiving distance of 200 mm, and a voltage of 30 kV, the novel nozzle achieves an average electric field intensity of 5.26 × 10⁶ V/m with a CV value of 6.93%. Metal 3D printing was used to create a new nozzle for electrospinning, which successfully produced stable multiple jets and increased nanofiber output.

## 1. Introduction

Nanofibers with great specific surface area, transverse to longitudinal ratio, radius of curvature, high porosity, and better mechanical properties have been widely studied and applied in many fields such as water treatment, biomedicine, textiles, filtration materials, energy field, and sensor technology in recent years [1,2,3]. Electrospinning is a simple and effective method to prepare nanofibers by stretching a polymer solution under the electric field generated by a high-voltage DC power supply [4,5,6]. To ensure the high-quality and high-volume production of nanofibrous membranes, the predominant electrospinning techniques currently employed are multi-needle electrospinning and needleless electrospinning [7,8]. Multi-needle electrospinning is mainly represented by W. Toniaszevvski et al. [9] who improve the yield by arranging multiple needles according to a certain layout, which has the advantages of controllable jet flow and low energy consumption. However, multi-needle electrospinning leads to an unstable electrospinning process and an uneven thickness of the fiber web from the middle to the two sides due to the phenomenon of mutual repulsion within the electric field between the adjacent needles, i.e., the End Effect [10]. Needleless electrospinning technology is mainly represented by spider nano-electrostatic spinning and bubble electrospinning technology, which use mechanical external force, electromagnetic force, gas expansion, and other roles to introduce the spinning liquid surface into the electric field and form multiple jets through the reorganization of the free-surface liquid [11,12]. To effectively increase the electric field strength while reducing the End Effect, D. Petras et al. proposed wave-shaped rotating electrodes [13]. They optimized the electric field by matching the electrode shape with the equipotential lines of the electric field and successfully prepared fibers with a diameter ranging from 100 to 500 nm. S. King et al. used rotating electrodes with conductive protrusions [14]. They optimized the electric field by optimizing the spacing and aspect ratio of the protrusions and used an external disk-shaped intensifier to balance the electric field intensity at both ends of the electrode. They successfully prepared fibers with a diameter of about 110 nm, which is comparable in quality to that of a single-needle system. Needleless electrospinning technologies can effectively enhance the production of nanofibers. Moreover, subsequent research on optimizing the shape of traditional drum-shaped emitters has been able to reduce the edge effect while strengthening the electric field intensity. However, due to the mostly open liquid supply, the solution is volatile, which leads to difficult control of the final fiber quality [15].

In response to the above problems, a multi-vane and multi-slit electrospinning nozzle, combining the respective advantages of multi-needle and needleless electrospinning, was proposed. Firstly, the multi-vane structure was derived according to Tate’s law in the principle of surface tension [16]. Increasing the contact area can increase the surface tension of the solution. Multiple outwardly extended vanes can increase the surface tension of the solution, so that the solution can spread better between the vanes, prolong the holding time of the solution, and improve the stability of electrospinning. Secondly, the multi-slit structure was obtained based on the capillary effect. When the liquid comes into contact with the solid, the surface tension tends to drive the liquid to move towards the narrow slits. Therefore, through the slit structure, the electrospinning solution can be evenly guided to the tip ends of each vane, so that multiple Taylor cones can be stably excited at multiple tips, improving the electrospinning efficiency. Furthermore, the liquid supply structure inside the novel nozzle is a semi-closed structure. In this way, while ensuring the stability of electrospinning and improving the efficiency, the liquid supply can also be relatively sealed, eliminating the influence of the open liquid supply in traditional needleless electrospinning. The novel electrospinning nozzles with different parameters were modeled by Solidworks 2018. A numerical simulation of the solution droplet free liquid surface was carried out by Ansys Fluent 2022 R1 software to demonstrate the advantages of the novel electrospinning nozzle in increasing the droplet holding time and the stability of the smooth liquid supply. The electrospinning system model was established, and the Comsol Multiphysics 6.0 software was used for the electric field simulation to study the effects of the number of vanes and the length of the vanes of the novel nozzle on the uniformity of the electric field.

## 2. Materials and Experiments

**Figure 1:**
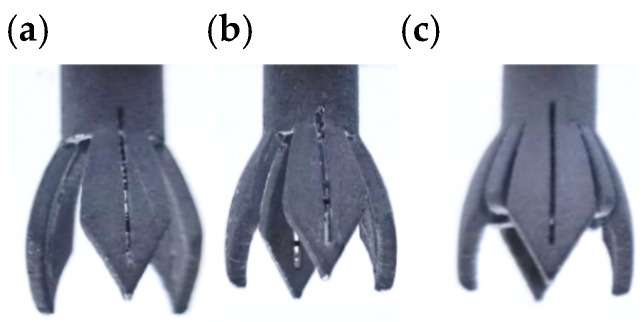
Metallic 3D-printed novel electrospinning nozzle. The outward spreading angles is 35° and the length of the vanes is 11 mm. (**a**) Nozzle with 3 vanes and without supported structure. (**b**) Nozzle with 4 vanes and without supported structure. (**c**) Novel nozzle with 4 vanes and supported structure.

The novel electrospinning nozzles with different parameters were fabricated separately by metal 3D printing, and the materials were all made of aluminum alloy (AlSiOMg), as shown in Figure 1. The electrospinning experiments were conducted using novel nozzles with varying parameters. The uniformity of the electric field at the novel nozzle tip, as observed in electric field simulations, was assessed by analyzing the coefficient of variation (CV) in the final fiber diameter produced. The CV value served as the standard for evaluating the consistency and uniformity of the fibers, which in turn reflects the stability and uniformity of the electric field at the novel nozzle tip during the electrospinning process.

### 2.1. Materials and the Solution

Polyacrylonitrile (PAN, average molecular weight 85,000, Shanghai Aladdin Biochemical Science and Technology Co., Ltd., Shanghai, China) and N-dimethylformamide (DMF, analytically pure, Tianjin Komeo Chemical Reagent Co., Tianjin, China) were used.

Preparation of the PAN solution with a 10% quality fraction: First, 90 g of DMF solvent was measured into a conical flask using an electronic balance. A magnetic stirrer was then added, and the flask was placed in a water bath for stirring. Next, 10 g of PAN powder was weighed and gradually poured into the stirred flask. The flask was sealed with plastic wrap to prevent water vapor from entering, and the water bath temperature was set to 60 °C. The mixture was stirred continuously for 5 h until the solution became clear, transparent, homogeneous, and stable. The solution was then left to stand until ready for use.

The syringe was connected to the novel electrospinning nozzle, and the prepared PAN spinning solution was then loaded into the syringe. The solution was spun using a homemade electrospinning device. The detailed specifications of the experimental equipment are provided in Table 1.

The positive high-voltage DC power supply was connected to the novel electrospinning nozzle, while the negative high-voltage DC power supply was connected to the receiving electrode and the base cloth (release paper). The positive high-voltage DC supply was set to 20 kV, and the negative supply to 10 kV. The receiving distance was 20 cm, with an electrospinning rate of 2 mL/h. Under the environmental conditions of a room temperature of 25 °C and a relative humidity of 50%, after 5 min of electrospinning, a PAN nanofiber membrane was obtained.

### 2.2. Morphological Observation and Fiber Diameter Prediction

A small section of the sample membrane was cut and placed flat on conductive adhesive after gold sputtering. The surface morphology was then observed using a Hitachi Flex SEM1000 Cold Field Scanning Electron Microscope, Hitachi, Tokyo, Japan. After completing the observations, the diameters of 50 randomly selected fibers from the SEM images were measured using Image Pro Plus 6.0 software. The average fiber diameter and the coefficient of variation (CV) were calculated, and a diameter distribution plot was created.

## 3. Method and Discussion

### 3.1. Structural Design of the Novel Electrospinning Nozzle

**Figure 2:**
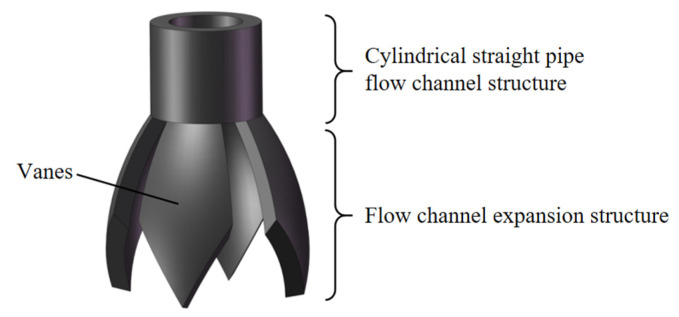
The schematic diagram of the structure of the new electrospinning nozzle. It includes cylindrical straight pipe flow channel structure and flow channel expansion structure.

As shown in Figure 2, the novel electrospinning nozzle is designed as semi-closed structure, with a cylindrical straight pipe flow channel at the top and a flow channel expansion structure at the bottom. The flow channel expansion structure is composed of multiple sheet-like components that spread outward at an angle, creating radial flow channels. Each sheet is referred to as a vane and is designed by Bézier curves. The tips of the vanes can generate multiple jets through electric field induction stably. During the electrospinning process, the nozzle is arranged vertically downward. The structure of multiple outwardly extended nozzle vanes increases the surface tension of the electrospinning liquid. While effectively preventing the accidental dripping of the electrospinning liquid, it mainly ensures that the electrospinning liquid can be smoothly supplied to the tips of the nozzle vanes, preventing accidental blockages inside the nozzle.

#### 3.1.1. Design of the Cylindrical Straight Pipe Flow Channel Structure

**Figure 3:**
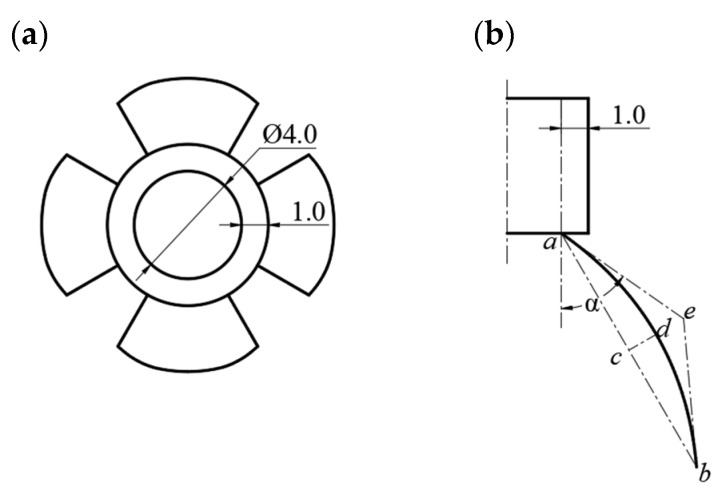
Key structural features of the novel electrospinning nozzle. (**a**) Cylindrical straight pipe flow channel structure diagram (inner diameter: 4 mm, wall thickness: 1 mm); (**b**) the unilateral vane curve is designed based on the Bézier curve.

Traditional needle electrospinning technology, which uses capillary needles with an inner diameter of 0.6 mm to 0.8 mm, results in low yield and is not suitable for high-viscosity polymer solutions. As shown in Figure 3a, to accommodate a wider viscosity range of the spinnable solution, the inner diameter of the cylindrical straight pipe flow channel is designed to be 4 mm. The thickness of the wall is 1 mm, and the overall length is 5 mm.

#### 3.1.2. Design of Vane Curves

The Bézier curve is a significant parametric curve in computer graphics [17].

(1) Basic mathematical principles of Bézier curve.

① First order Bézier curve. Given two points, *P*_0_ and *P*_1_, which form a line segment, a point *P* on the line segment can be determined through linear interpolation based on a parameter *t*. The trajectory of *P* describes the first-order Bézier curve, as given by Equation (1):

(1) $$P\left(t\right)=P_{0}+\left(P_{1}-P_{0}\right)t=\left(1-t\right)P_{0}+tP_{1},t\in \left[0,1\right]$$

where *P*(*t*) is the coordinate with respect to the *t* parameter; *P*_0_ and *P*_1_ are the two given fixed points; and *t* is the parameter.

② Quadratic Bézier curve. Given three distinct points *P*_0_, *P*_1_, and *P*_2_, connect them sequentially with line segments. By applying linear interpolation based on the first-order Bézier equation, a quadratic Bézier curve is generated, as described by Equation (2):

(2) $$B\left(t\right)={\left(1-t\right)}^{2}P_{0}+2t\left(1-t\right)P_{1}+t^{2}P_{2},t\in \left[0,1\right]$$

where *B*(*t*) is the coordinate with respect to the *t* parameter; *P*_0_, *P*_1_, and *P*_2_ are the three fixed points that are given non-collinear; and *t* is the parameter.

(2) Graphical representation of Bézier curves.

① First order Bézier curve. Given 2 points, *P*_0_ and *P*_1_, it can form a straight line.

② Quadratic Bézier curves. Given 3 points, *P*_0_, *P*_1_, and *P*_2_, it can form a quadratic curve; the principle is to make the curve approximate the triangle formed by the points *P*_0_, *P*_1_, and *P*_2_ so that the curve can express a shape with a certain arc.

(3) Design of vane curve.

A quadratic Bézier curve is used to design the vane curve of the flow channel expansion structure, as shown in Figure 3b. Observed from the model cross-section, the unilateral vane curve is the curve $\overset{⏜}{ab}$. For ease of calculation, take the straight-line distance between the two ends of the curve as the length of the vane ($l_{ab}$), and the geometric relationship has the constructive line *ae* and *eb* with equal distance ($l_{ae}=l_{eb}$).

Design of curved arcs: point *c* is the midpoint of ($l_{ab}$), and the curved arc height ($l_{cd}$) is such that the ratio $l_{cd}:l_{ab}=1:10$.

Design of vane curve opening angle: from the quadratic Bézier curve theory, the construction line *ae* tangent to the curve $\overset{⏜}{ab}$, by taking the construction line *ae* and the angle of the vertical line for the vane curve opening angle *α*, can be seen. Finally, use Solidworks 2018 software to model the vanes. Apply the rotary thickening command to create the main body of the vanes and then use the cut command to shape the vane tips with a 60° angle.

### 3.2. Fluid Simulation and Optimization of the Novel Electrospinning Nozzle

The vanes parameters of the flow channel expansion structure of the novel electrospinning nozzle include the vanes opening angle, the number of vanes, and the length of the vanes. In the electrospinning process, the size of the liquid surface and the ability to hold electrospinning droplets have a significant impact on yield improvement. A larger liquid surface enhances yield, while a longer droplet holding time contributes to the stability of the electrospinning process. The process from the beginning of the formation of a droplet to its falling is called the “holding time” of the droplet. When the electrospinning solution flows through the cylindrical straight pipe flow channel of the novel nozzle, it spreads along multiple vanes, causing the liquid surface to gradually expand. This increases the contact area between the electrospinning liquid and the novel nozzle, enhancing the surface tension of the liquid along the inner wall and thereby improving the droplet-holding time. The following is a numerical simulation of the free liquid surface of droplets in the novel electrospinning nozzle using Ansys Fluent 2022 R1 software. The state showing the maximum liquid surface spread and the longest droplet-holding time is selected as the optimal steady state for studying the spreading behavior and flow dynamics of the electrospinning liquid along the inner wall of the novel nozzle.

#### 3.2.1. Numerical Simulation of Fluid Spreading in the Novel Electrospinning Nozzles

The numerical simulation method is a comprehensive process, which is mainly divided into software pre-processing, solver, and result post-processing. Several key steps are explained and analyzed in detail below.

(1) Geometric model import and meshing

**Figure 4:**
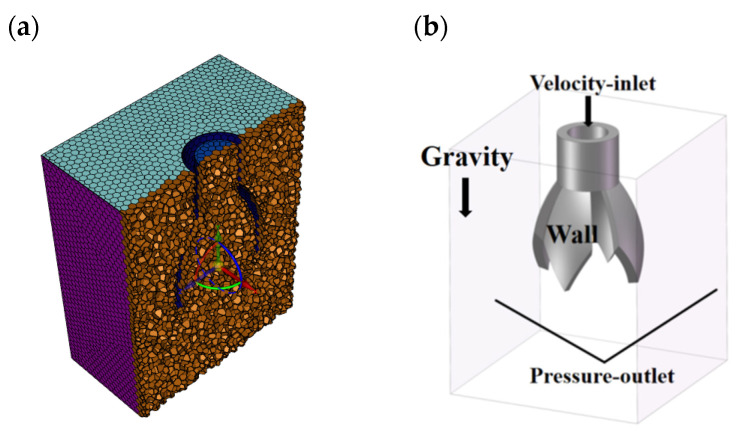
Mesh generation and numerical simulation of the novel nozzle. (**a**) Cross-section of the novel nozzle mesh (minimum cell size: 0.2 mm, average cell size: 0.95 mm). (**b**) Numerical simulation model showing the flow field space (20 mm × 20 mm × 25 mm) and boundary conditions (velocity inlet: 1 × 10⁻^3^ m/s, pressure outlet: 0.1 MPa) used to analyze droplet holding behavior and liquid surface dynamics.

The novel nozzle models with vane opening angles of 25°, 30°, 35°, 40°, and 45° were built using Solidworks 2018 software. The cylindrical straight pipe flow channel has a length of 5 mm, an inner diameter of 4 mm, a wall thickness of 1 mm, a vane length of 10 mm, and a vane opening angle α of variable. The geometric model generated in Solidworks 2018 is imported into Spaceclaim 2022 R1 for volume extraction. It is then transferred to Fluent Meshing 2022 R1 for meshing using hexahedral elements, with a minimum cell size of 0.2 and an average cell size of 0.95. The resulting mesh is shown in Figure 4a.

(2) Model selection and boundary condition setting scheme.

A cube with a length and width of 20 mm and a height of 25 mm is established as the flow field space outside the novel nozzle, and the novel nozzle flow channel and flow field space are shown in Figure 4b.

The numerical simulation process of Ansys Fluent software is based on the pressure solver using the VOF multiphase flow model, laminar flow mode, gravity setting, and transient solution; the main phase is air and the second phase is set to be a non-Newtonian fluid substance, with the fluid properties defined with reference to the properties of a PAN solution with a solution mass percent concentration of 10 per cent. The inlet uses the velocity inlet and the inlet velocity is set to 1 × 10^−3^ m/s. The six faces of the external flow field space are all outlets, and the outlets are pressure outlets; the outlet pressure is standard atmospheric pressure = 0.1 MPa, and the other wall surfaces are wall boundary conditions.

(3) Analysis of calculation results

**Figure 5:**
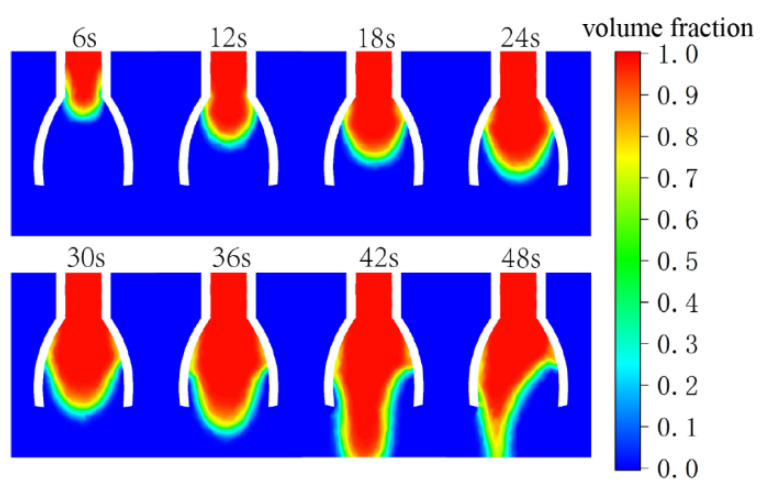
The distribution of fluid volume fraction inside the novel electrospinning nozzle over time. The cloud image illustrates the spreading behavior of the electrospinning solution (10% PAN) along the vanes (opening angle: 35°).

Taking the novel nozzle vane opening angle of 35° as an example, the trend of the second phase volume fraction cloud image is shown in Figure 5.

It can be observed that the liquid is fully spread out at around 36 s, which is the optimal state of the liquid level, and then it stops spreading and begins to gradually form raised droplets that eventually fall. In addition, as the droplets gradually form, they tend to deviate and fall to one side. This is because the electrospinning flow rate is relatively slow, and the flow volume is very small. Under the action of the surface tension of the liquid, the fluid at the outlet will form fluctuations or deviations. This is a normal phenomenon and will not affect the experimental results.

#### 3.2.2. Impact of the Novel Nozzle Vane Opening Angle on Electrospinning Liquid Surface Spreading

**Figure 6:**
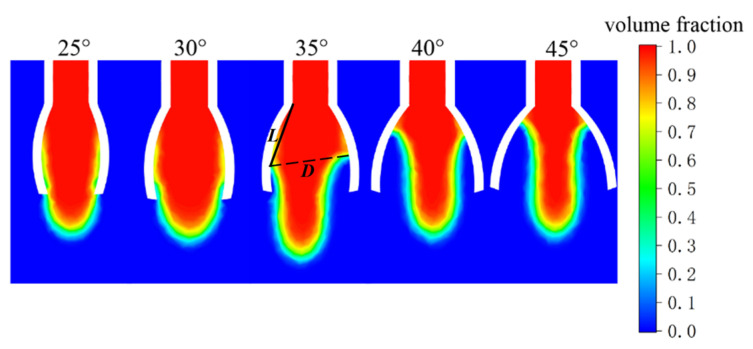
Distribution of fluid volume fraction at full spreading state for vane opening angles ranging from 25° to 45°.

Following the above process, the fluid spreading of the novel nozzles with 25°, 30°, 35°, 40°, and 45° vane opening angles was simulated, and the distribution of the fluid volume fraction at the moment when the fluid is fully spread is shown in Figure 6. Taking the vane opening angle of 35° as an example, the solid straight line *L* represents the spreading length of the liquid, and the dashed straight line *D* represents the maximum diameter of the liquid surface. The measured results of the spreading length of the fluid and the maximum liquid surface diameter are listed in Table 2. According to the data in Table 2, the maximum spreading length of the fluid decreases as the opening angle of the novel nozzle vanes increases, with none exceeding 9 mm. Additionally, the maximum diameter of the liquid surface varies with the vane opening angle, showing an initial increase followed by a decrease from 25° to 45°. When the novel nozzle vanes have an opening angle of 35°, the maximum diameter of the liquid surface reaches 8.966 mm. During the electrospinning process, a larger liquid spreading surface inside the nozzle can facilitate the diversion effect of multiple slits. This enables the continuous delivery of the spinning solution to the tips of the vanes, providing a guarantee for improving the efficiency and stability of electrospinning. Therefore, the opening angle of the vanes should be chosen as 35°.

#### 3.2.3. Impact of the Novel Nozzle Vane Mid-Slit and Support Structure on Droplet Diversion and Holding

**Figure 7:**
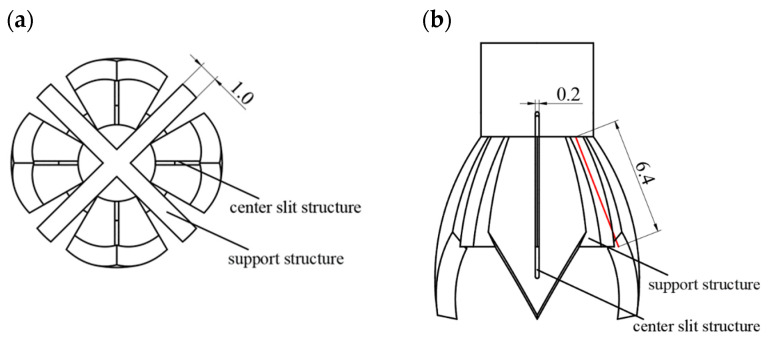
Structural design of the novel electrospinning nozzle with center slit and support structure. (**a**) Top view showing the radial arrangement of vanes (opening angle: 35°) and the central slit (width: 0.2 mm) for precise liquid diversion. (**b**) Main view highlighting the support structure (length: 6.4 mm), which increases liquid contact area and enhances droplet holding time. The red line in the figure represents the droplet spreading length when the vane is opened at 35° as shown in Table 2.

As the liquid spreads to its maximum extent along the novel nozzle vanes, the liquid surface gradually forms a bulging droplet. Over time, the weight of the droplet accumulates, eventually causing it to detach and fall. The process from the beginning of the formation of a droplet to its falling is called the “holding time” of the droplet. The longer the holding time, the less likely the droplets are to drip, thus the more favorable the stability of the electrospinning. At the same time, due to the tip effect of the nozzle, during electrospinning, the charges mainly accumulate at the tip ends of the vanes. The electric field intensity is the highest at the tips. Therefore, precisely guiding the liquid to the tips is more conducive to the formation of the Taylor cone. Therefore, the following investigation focuses on the effects of the internal support structure and the central slit design of the novel nozzle on droplet holding and drainage. As shown in Figure 7, similar to the capillary action observed in a fountain pen nib and its slit, the liquid moves due to surface tension through microscopic holes, capillaries, or tiny channels. This capillary effect causes the ink to be drawn to the front of the nib, where it is transferred to the iridium particles to complete the writing process. Therefore, a slit of 0.2 mm in width is first set in the middle of the vanes so that the solution inside the novel nozzle is accurately diverted to the tip of the vanes at the end of the novel nozzle.

Design of the novel nozzle support structure based on the longest spreading length of the liquid when the vanes are open at 35° in Table 2. As shown in Figure 7a, the support structure consists of multiple support plates arranged such that two vertical support plates intersect at intervals between each pair of vanes, depending on the number of novel nozzle vanes. As shown in Figure 7b, the red line represents the maximum length of the spreading of the liquid, and the height of the support structure is level with the maximum spreading length of the liquid. This way the support structure increases the contact area of the liquid, thereby increasing the surface tension of the liquid and enhancing the effect of holding the liquid.

**Figure 8:**
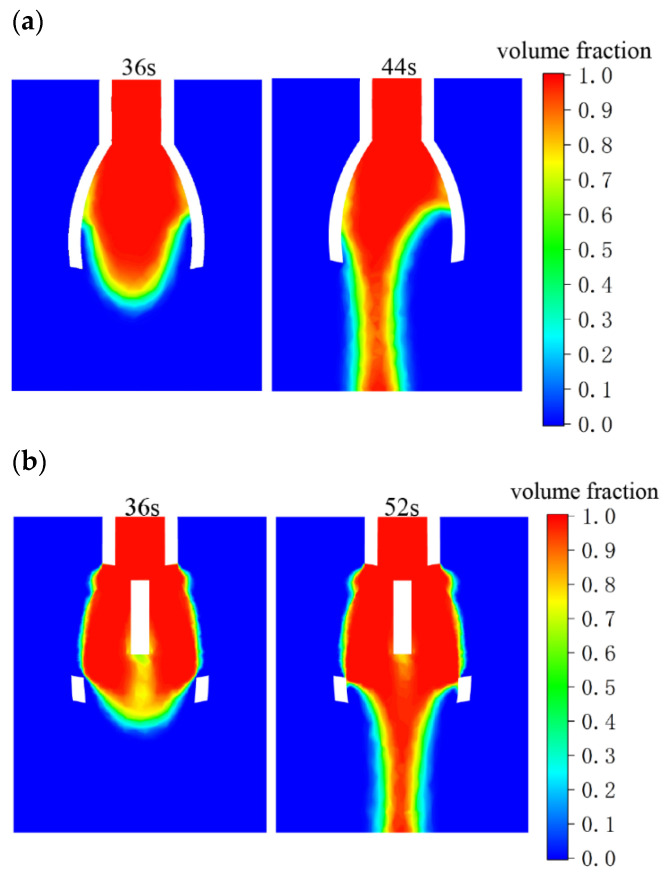
Comparison of droplet holding time between novel nozzles with and without central slit and support structure. (**a**) Nozzle without support structures: droplet holding time limited to 8 s due to weak surface tension. (**b**) Nozzle with support structures: holding time doubled to 16 s via increased liquid contact area, ensuring stable electrospinning.

Finally, the novel nozzle with the central slit and support structure is modeled using Solidworks 2018 software, and fluid simulation is performed using Ansys Fluent 2022 R1 software. The simulation results are shown in Figure 8, where the liquids from both novel nozzles are fully spread out at about 36 s, after which the droplets are gradually formed. Comparing the droplet holding times for the two novel nozzles, it is observed that the novel nozzle without a central slit and support structure sees the droplet drop at 44 s, with a holding time of 8 s. In contrast, the novel nozzle with a central slit and support structure allows the droplet to remain for 16 s before dropping at 52 s, indicating a significant improvement in droplet holding capability. With the central slit and support structure, the novel nozzle not only prevents liquid from dripping during supply but also utilizes the capillary effect to facilitate the transport of the spinning liquid, ensuring a continuous and smooth supply for electrospinning.

#### 3.2.4. Droplet Holding Experiments in Novel Electrospinning Nozzle

Droplet experiments are conducted on novel electrospinning nozzles with both supported and unsupported structures, which have been optimized through fluid simulation. The cylindrical straight pipe flow channel of the novel nozzle has a length of 5 mm, the vanes are each 10 mm long, there are 4 vanes, and the vane opening angle α is 35°. A micro syringe pump is used to supply the liquid, which is a 10% PAN solution.

The pump is set to deliver the liquid at a rate of 1 × 10^−3^ m/s, based on the numerical simulation results from the previous section. A camera records the spreading of the solution along the novel nozzle vanes, measuring the time from the moment the solution is fully spread to the formation of droplets and their subsequent fall. This duration is referred to as the droplet holding time.

**Figure 9:**
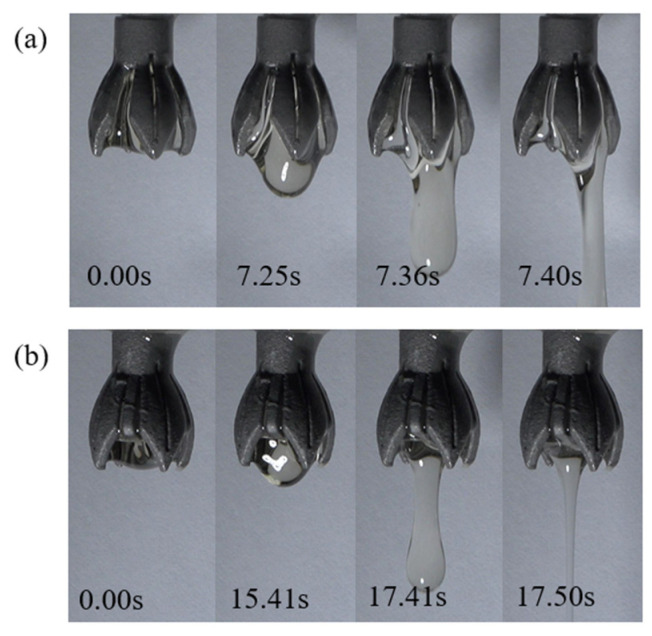
Droplet formation process in novel electrospinning nozzles with and without support structure. (**a**) Nozzle without support structures (4 vanes): droplet holding time limited to 7.4 s. (**b**) Nozzle with support structures (4 vanes, 11 mm vane length): holding time extended to 17.5 s.

As shown in Figure 9, when the electrospinning solution spreads along the vanes to its limit, droplets begin to form at the center of the liquid surface. This process is relatively slow. As the mass of the droplets increases, they eventually overcome the surface tension of the liquid and fall due to gravity. From the time recorded by the camera, it can be found that the droplet holding time of the novel nozzle without support structure is 7.40 s, and the droplet holding time of the novel nozzle with support structure is 17.50 s. Consistent with the simulation results from the previous section, the support structure significantly impacts droplet holding, effectively enhancing the stability of the electrospinning process.

### 3.3. Simulation and Optimization of Electric Field in the Novel Electrospinning Nozzle

In the electrospinning process, the structure of the novel nozzle significantly influences both the magnitude and uniformity of the electric field, which directly impacts the stable formation of the Taylor cone and the quality of the final fiber [18]. This section uses Comsol Multiphysics 6.0 software to simulate the electric field in novel electrospinning nozzles with various structural parameters. The simulation aims to observe the average electric field intensity and its distribution, identifying the structural parameter that yields the highest average electric field intensity and the most uniform electric field.

#### 3.3.1. Modeling of the Electrospinning System

**Figure 10:**
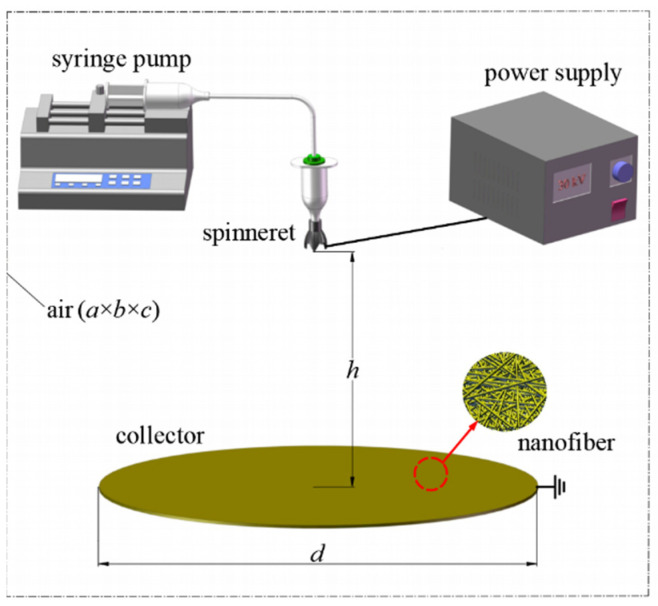
Electrospinning system model for electric field simulation.

The model of the electrospinning system is established as shown in Figure 10, including the spinning emitter, the fiber receiving the electrode (disk shape), and air [19]. As shown in Figure 10, *a*, *b* and *c* are the length, width, and height of the air cube, with values of 600 mm each. *d* denotes the diameter of the receiving electrode, which is 300 mm, and *h* is the distance from the emitter to the receiving electrode, set at 200 mm. During the simulation, a positive voltage of 30 kV is applied to the novel nozzle of the emitter, while the receiving electrode is grounded.

Based on the model parameters shown in Figure 10 and the fundamental principles of electrostatic fields, Comsol Multiphysics 6.0 software was used to simulate the electric field. The study focused on examining how the number of vanes and the length of the vanes in the novel electrospinning nozzle affect the electric field intensity and its distribution.

#### 3.3.2. Effect of the Number of the Novel Nozzle Vanes on Electric Field

To examine the effect of different numbers of vanes on the electric field in novel electrospinning nozzles, the model is simplified by omitting the support structure in this part of the study. The support structure will be incorporated once the optimal number of vanes is determined.

With the requirement that the liquid is fully spread, the vane length is set to an initial value of 10 mm. Models are created with 2, 3, 4, 5, and 6 vanes, respectively. These models are then imported into the electrostatic field module of Comsol Multiphysics 6.0 for finite element simulation to determine the electric field intensity at the tip of the vanes for different configurations of the novel electrospinning nozzle. Calculate the average electric field intensity and the coefficient of variation (CV) for each configuration. Then, use Origin 2021 software to plot the average electric field intensity, CV values, and their distribution curves. The coefficient of variation is calculated as Equation (3):

(3) $$\mathrm{CV}=(S/\bar{X})\times 100\%$$

where S is the standard deviation and $\bar{X}$ is the mean.

The average electric field intensity and its distribution.

**Figure 11:**
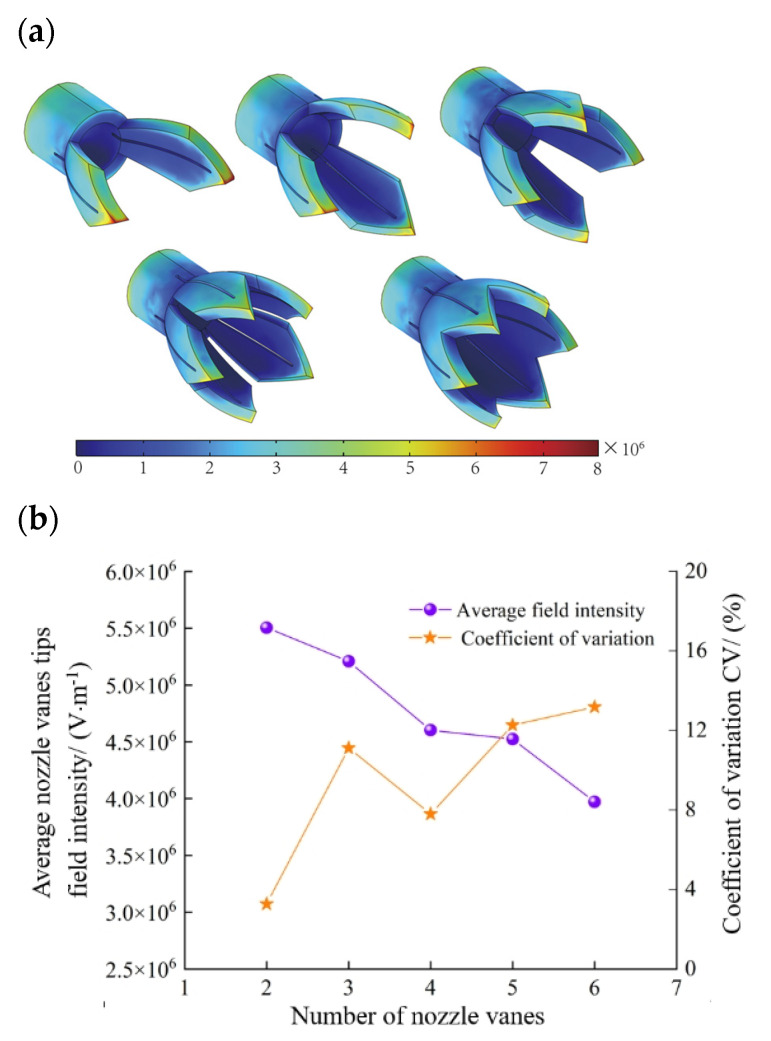
The effect of the number of novel nozzle vanes on the electric field. (**a**) Electric field distribution cloud images of novel electrospinning nozzle with a different number of vanes. (**b**) Electric field intensity and CV value of the novel electrospinning nozzle with a different number of vanes.

Figure 11a illustrates the electric field cloud for the novel nozzles with different numbers of vanes. It is easy to see that as the number of vanes increases, the intensity of the electric field at the tip of the novel nozzle vanes decreases. The specific data are shown in Figure 11b, where the average value of the vanes tip field intensity decreases from 5.50 × 10^6^ V/m to 3.97 × 10^6^ V/m with the increase in the number of vanes. This is because as the number of vanes increases, the Coulomb repulsive interactions between the tips become more pronounced, reducing the induced electric field at the tips. Therefore, having too many vanes is not advisable. In the electrospinning process, the coefficient of variation (CV) of the electric field intensity reflects the uniformity of the electric field. A smaller CV value indicates a more uniform electric field. To ensure stable electrospinning, it is important to focus on minimizing the CV value, provided that the average electric field intensity remains relatively consistent.

Figure 11b, on the right axis, displays the coefficient of variation (CV) of the field intensity for different numbers of vanes. It can be observed that for 2 and 4 vanes, the CV values are 3.27% and 7.80%, respectively, indicating that the tip field intensity CV is relatively small, remaining below 10%. At the same time, the electric field intensities corresponding to 2 and 4 vanes are also relatively higher, being 5.51 × 10^6^ V/m and 4.60 × 10^6^ V/m, respectively. Therefore, the parameter for the optimal number of vanes should be among these two values. Considering the droplet holding time of the electrospinning solution, when the number of vanes is 2, the solution is prone to droplets, which is not favorable to the generation of the Taylor cone and the electrospinning instability. Therefore, the optimal parameter for the number of nozzle vanes should be set to 4. It features a relatively high electric field intensity and good droplet-holding properties. More importantly, the CV value is also lower.

The following section takes the novel electrospinning nozzle with 4 vanes as an example and considers the length of the vanes and further studies methods to improve the electric field intensity of the novel electrospinning nozzle with 4 vanes and enhance the uniformity of the electric field.

#### 3.3.3. Effect of the Length of the Novel Nozzle Vanes on Electric Field

The number of novel nozzle vanes has been determined to be 4, so the support structure is set up as two support plates placed crosswise in the interval of every two vanes. For the 4-vane electrospinning novel nozzle with support, the effect of vane length on the electric field is explored. The vane length is set to 7 mm, 8 mm, 9 mm, 10 mm, 11 mm, 12 mm, and 13 mm. The electric field is then simulated for each length, and the simulation results provided the field intensity values at the tips of the novel electrospinning nozzle vanes. The average electric field intensity and the coefficient of variation (CV) of the electric field intensity are subsequently calculated.

**Figure 12:**
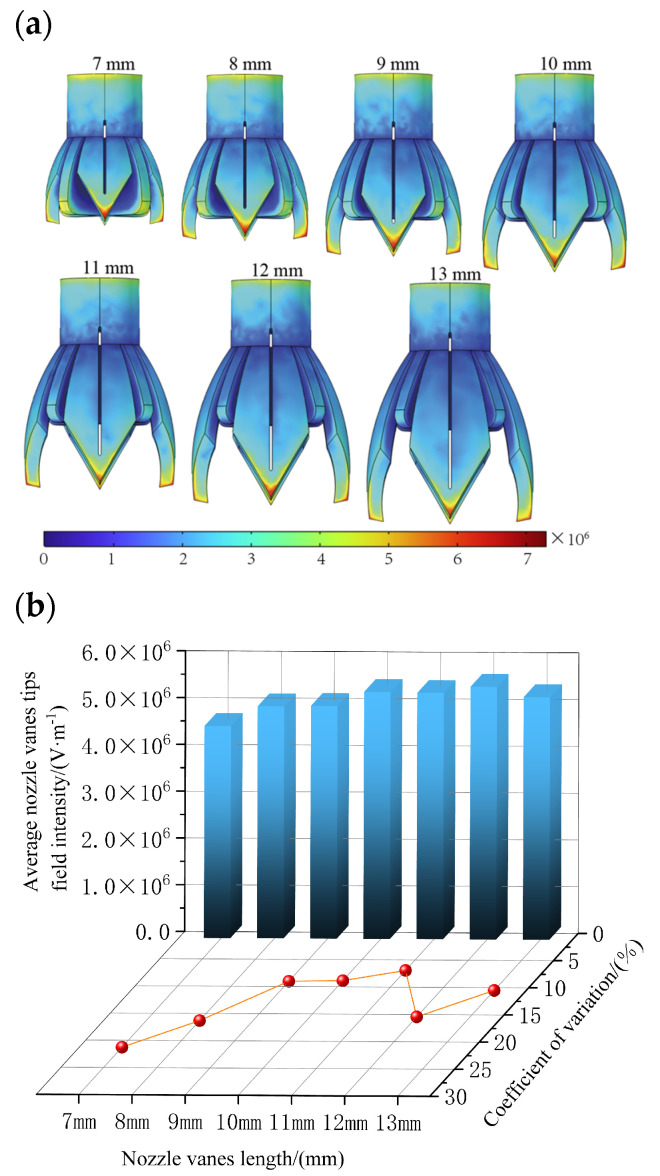
The effect of novel electrospinning nozzle with different vanes length on electric field. (**a**) Electric field distribution cloud images of novel electrospinning nozzle with different vane lengths. (**b**) Average electric field intensity and CV values of electric field intensity of novel electrospinning nozzle with different vane lengths.

Figure 12a shows the electric field cloud of the novel electrospinning nozzle with different vane lengths, and Figure 12b shows the average value of the electric field intensity with field intensity CV for different vane lengths.

From Figure 12b, it can be observed that as the vane length increases from short to long, the electric field intensity at the novel nozzle tip initially rises slowly from 4.53 × 10^6^ V/m to 5.40 × 10^6^ V/m, and then decreases to 5.18 × 10^6^ V/m. This behavior is attributed to the influence of the support structure, which, when the vanes are shorter, is positioned closer to the tip. This proximity affects the electric charge distribution at the tip, resulting in lower field intensity. Then, as the vanes increase in length, the tip of the vanes moves away from the support structure and the tip is closer to the receiving pole; the charge will again accumulate more at the tip, resulting in an increase in the average field intensity at the tip. However, as the vane length continues to increase, the surface area of the novel nozzle also grows, which reduces the charge density per unit area. This ultimately leads to a decrease in the field intensity at the tip of the vanes. Finally, considering the coefficient of variation (CV) value alongside the field intensity, and noting that the field intensity values are relatively similar, a vane length of 11 mm is preferred. This length can provide the smallest CV value, which is 6.93%, and at the same time, the average electric field intensity is 5.26 × 10⁶ V/m. In this way, while ensuring a certain electric field intensity, it can also ensure that the droplets can be fully spread and formed normally. Therefore, the novel electrospinning nozzle should use vanes with a length of 11 mm.

## 4. Results and Discussion

### 4.1. Experimental Analysis of Electrospinning

**Figure 13:**
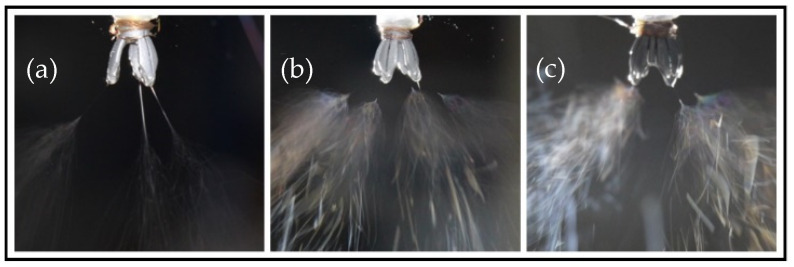
Comparison of electrospinning performance among three nozzles with different structural parameters. (**a**) Nozzle with 3 vanes and without supported structure. (**b**) Nozzle with 4 vanes and without supported structure. (**c**) Novel nozzle with 4 vanes and supported structure.

Figure 13 illustrates the electrospinning process for three types of novel nozzles: (a) a 3-vane unsupported structure novel nozzle, (b) a 4-vane unsupported structure novel nozzle, and (c) a 4-vane supported structure novel nozzle (with vanes length of 11 mm). It can be observed that as the electrospinning solution flows through the cylindrical straight pipe flow channel, the center slit diversion causes a uniform distribution of the solution at the tips of each vane. This configuration enables all types of novel nozzles to generate multiple electrospinning jets.

In terms of droplet holding, Figure 13a,b reveal that novel nozzles without a support structure fail to retain the solution droplets on the vanes for an extended period, resulting in premature droplet detachment. Consequently, these novel nozzles only produce jets from residual droplets, leading to unstable and unsustainable electrospinning.

In contrast, for the nozzle with a support structure depicted in Figure 13c, thanks to the reinforcement of the support structure inside the nozzle, the solution droplets can be stably held within the vanes for an extended period. It continuously reserves the solution for the drainage of the central slit, and the dispersion effect of the central slit is even more pronounced. Small solution droplets can be distinctly observed to be hanging at the tips of the vanes. The electrospinning jet is stable and uniform, which is basically consistent with the previous theory.

### 4.2. Analysis of Fibrous Membrane Surface Morphology and Fiber Diameter Distribution

**Figure 14:**
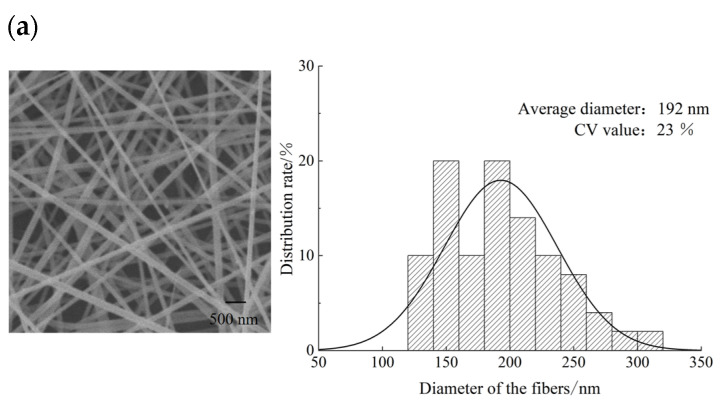

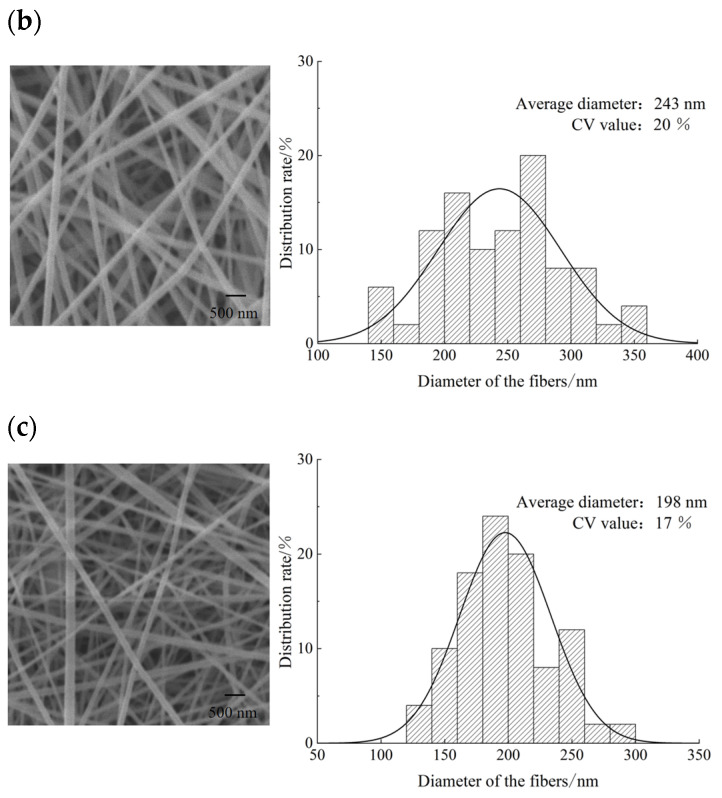
Sem photo and diameter distribution of nanofiber membrane. (**a**) Fiber electron micrographs and diameter distributions of novel nozzles with 3 vanes unsupported structures. (**b**) Fiber electron micrographs and diameter distributions of novel nozzles with 4 vanes unsupported structures. (**c**) Fiber electron micrographs and diameter distributions of novel nozzles with 4-vane supported structures.

The observation of the surface morphology of the fibrous membrane by the Hitachi Flex SEM1000 Hitachi Cold Field Scanning Electron Microscope with fiber diameter distribution is shown in Figure 14. Diameter measurements using Image Pro Plus 6.0 software revealed that the coefficient of variation (CV) for the fiber diameter of the electrospinning fibers from the 4-vane (11 mm length) novel nozzle with a support structure was 17%. This is significantly better compared to the other two novel nozzle types, which aligns with the CV values of the tip electric field intensity from the previous electric field simulations. This indicates that a more homogeneous electric field at the novel nozzle tip results in a more uniform fiber diameter distribution.

From the analysis, it is evident that a higher electric field intensity tends to produce fibers with a finer diameter. The simulation results indicated that the average electric field intensity at the tip of the 3-vane unsupported structure novel nozzle was significantly higher than that of the 4-vane unsupported structure novel nozzle. Consequently, in the fiber diameter distribution graph from the final experiment, the average diameter of fibers produced by the 3-vane unsupported structure novel nozzle (192 nm) was noticeably smaller than that of fibers from the 4-vane unsupported structure novel nozzle (243 nm).

## 5. Conclusions

In this paper, a novel nozzle electrospinning method is proposed. Through numerical simulation and experimental validation, the study investigates the novel nozzle structure, holding of the novel nozzle liquid, and optimization of electric field uniformity. These improvements significantly address common issues in multi-needle electrospinning, such as clogging, End Effects between needles, and challenges in needleless electrospinning related to liquid volatility and uncontrollable Taylor cone positioning. The specific conclusions are as follows:

(1) Combined with multiple fluid spreading simulations, it was first determined that the optimal fluid holding effect was achieved when the vanes of the novel nozzle were opened at an angle of 35°; in order to strengthen the precise liquid diversion and droplet holding, the middle of the novel nozzle vane is set with a center slit structure, and the internal support structure is set to prevent dripping while ensuring the smooth supply of electrospinning solution and improve electrospinning stability; the optimized novel nozzle is subjected to droplet experiments, and the droplet holding time of the novel nozzle with a center slit and support structure is 17.50 s, which is similar to that of the fluid spreading simulation results, and the support structure has a significant effect on the increase in droplet holding, indicating that this method can effectively improve the stability of the novel nozzle during electrospinning.
(2) After several sets of electric field simulation and comparison studies, it was finally determined that the electric field distribution was most uniform when the number of novel nozzle vanes was four and the length of vanes was 11 mm. In the electric field simulation of the electrospinning system, the average value of the electric field intensity was 5.26 × 10^6^ V/m with a CV value of 6.93% when the receiving distance is 200 mm and the voltage was 30 kV, and the electric field intensity at the electrospinning site is high and uniformly distributed; the final optimized novel electrospinning nozzle was used as the emitter for the electrospinning experiments, in which each vane tip was able to produce a continuous and stable counterpart jet with a large electrospinning yield, and the final fibers produced had an average diameter of 198 nm with a CV value of 17%.
(3) It should be taken into account that the current experimental study is primarily based on a 10% PAN solution frequently used in electrospinning. Other solutions (e.g., PVA, hydrophobic PVDF, et al.) can be used to further validate this electrospinning process of the novel nozzle in the following research. Additionally, the manufacturing cost of metal 3D-printed nozzles is relatively high, and the cost of mass production can be reduced by making molds in the future.

## Figures and Tables

**Table 1:** Experimental instruments.

Instruments	Type	Source
Spinning device	The novel electrospinning nozzle	In-house
DC high-voltage power supply	DW-P/N603	Tianjin Dongwen High Voltage Power Supply, Ltd., Tianjin, China
Metal halide lamp	70W	Xincheng Lighting, Ltd., Huzhou, China
Motor agitator	DF-101S	Gongyi Yuhua Instrument Co., Ltd., Zhengzhou, China
Thermostat water bath	HH-4	Kexi Instrument, Ltd., Changzhou, China

**Table 2:** The spreading length of the fluid and the maximum liquid surface diameter.

Angle of Opening *α*/°	Spread Length *L*/mm	Maximum Diameter *D*/mm
25°	9.864	5.241
30°	9.438	7.500
35°	6.397	8.966
40°	4.421	8.261
45°	3.181	7.637

## Data Availability

Data are contained within the article.
